# A spatial analysis of geographic variation and factors associated with hospitalization for bacterial pneumonia in Korea

**DOI:** 10.1186/s12890-019-0798-6

**Published:** 2019-02-20

**Authors:** Agnus M. Kim, Sungchan Kang, Jong Heon Park, Tae Ho Yoon, Yoon Kim

**Affiliations:** 1 Department of Health Policy and Management, Seoul National University College of Medicine, 103 Daehak-ro, Jongno-gu, Seoul, Republic of Korea; 2 Graduate School of Public Health, Seoul National University, Seoul, Republic of Korea; 3 National Health Insurance Service, Wonju, Republic of Korea; 4 Department of Preventive and Occupational Medicine, School of Medicine, Pusan National University, Pusan, Republic of Korea

**Keywords:** Pneumonia, Bacterial pneumonia, Hospitalization, Geographic variation, Spatial analysis, Spatial regression, Spatial autocorrelation, Hospital bed supply, Regional deprivation, Primary care, Korea

## Abstract

**Background:**

The incidence of pneumonia in Korea started to increase in the 1990’s after a period of decrease and stabilization, and the mortality and hospitalization rates for pneumonia in Korea are alarmingly high. This study was performed to examine geographic variation and factors associated with hospitalization for bacterial pneumonia in Korea.

**Methods:**

Data were acquired from the inpatient claims of the 2015 period of the National Health Insurance Service. The age- and sex-standardized hospitalization rates for bacterial pneumonia were calculated for three age groups. Geographic variation was measured with the coefficient of variation, the ratio of the 90th to the 10th percentile of the distribution of rates, and the systematic component of variation. Considering the results of Moran’s I statistic which suggested spatial autocorrelation, we estimated spatial regression models using spatial error models.

**Results:**

The hospitalization rate for bacterial pneumonia was 79.1 per 10,000 population, and the rate was the highest in the age group 0–14 at 325.3, and it was 161.5 among the elderly. The geographic variation statistics showed high variation with the coefficient variation at 0.6. The deprivation score showed positive associations, and the number of primary care physicians had a negative association with the hospitalization rates across all age groups but the age group 0–14. The number of beds in hospitals with less than 300 beds had a positive association with the hospitalization rates for bacterial pneumonia, and the impact was the strongest in the age group 0–14.

**Conclusions:**

The present study shows that pneumonia can be a major public health issue even in a developed country. Socioeconomic conditions can still be a concern for pneumonia in developed countries, and the role of primary care physicians in preventing hospitalization for bacterial pneumonia needs to be recognized. Most of all, the strong impact of hospital beds on the hospitalization rates for pneumonia, especially for the children, should be addressed. High disease burden of pneumonia in Korea can partly be attributable to oversupply of hospital beds. These factors should be taken into consideration in establishing policy measures for the rise in pneumonia.

**Electronic supplementary material:**

The online version of this article (10.1186/s12890-019-0798-6) contains supplementary material, which is available to authorized users.

## Background

Pneumonia is the world’s leading cause of death [1–3] and the single largest infectious cause of death among children [4]. While the burden of pneumonia is heavier in areas with low socioeconomic conditions [5], pneumonia is also a public health concern in developed countries as shown in the case of the US, where it is the most common cause of hospitalization, and of some European countries [3, 6–8]. Considering that pneumonia is deemed a preventable condition in terms of both incidence and hospitalization due to the applicability of vaccine and outpatient care manageability [9], it is important to analyze factors which keep pneumonia prevalent. In particular, given its nature as an infectious disease, the ever high prevalence of pneumonia in developed countries, where conditions regarding nutrition, sanitation, vaccination and medical service provision are more favorable than developing countries, needs to be analyzed from a broader perspective. High variation of mortality from pneumonia among developed countries [9] also suggests that the prevalence of pneumonia cannot be simply explained by the ageing of the population or population health status.

Korea is a developed country with a high mortality rate for pneumonia at 3.8 deaths per 100,000 as of 2017 [10]. Korea took a rapid path from an underdeveloped to a developed country within a few decades. Along with the economic growth and overall improvement in living conditions, the disease patterns in Korea changed with the marked rise in deaths from cardiovascular disease and cancers, while the proportion of pneumonia as a cause of death continued to decrease in the latter half of the twentieth century [11–13]. However, since the mid 1990’s, deaths due to pneumonia have continued to increase, which moved pneumonia from the tenth to the fourth leading cause of deaths during the last 10 year period [14]. This phenomenon can be partly attributed to the change in the demographic structure which involved an increase in the elderly population. However, the mortality rate due to pneumonia within the elderly population increased sharply as well. During the last 20 years, the mortality rate due to pneumonia among those 65 and over increased around five-fold [12] while the proportion of the population of them increased about 40% points [15, 16]. This suggests that there are factors that increased the incidence of pneumonia regardless of the change in the population structure.

The factors for the increase in the incidence of pneumonia can be analyzed from various facets, and a cross-sectional analysis of geographic variation in its rates can be especially useful to identify factors concerning health care supply and socio-economic conditions of a region. This study was performed to examine geographic variation and factors associated with hospitalization for bacterial pneumonia. First, we calculated the hospitalization rates due to bacterial pneumonia in 252 districts in Korea for the entire population and three age groups, and we investigated variations of these rates. Second, we analyzed the regional factors for the hospitalization rates for bacterial pneumonia using spatial regression analysis.

## Methods

### Data

We used a dataset of the inpatient claims in Korea for the 2015 period obtained from the National Health Insurance Service, which covers the entire population of Korea. To identify hospitalizations for bacterial pneumonia, we used the definition of ambulatory care sensitive conditions presented by the Institute of Medicine and the Agency for Healthcare Research and Quality [17, 18]. The International Classification of Diseases, Ninth Revision, Clinical Modification (ICD-9-CM) Codes for bacterial pneumonia were 481 (bacterial pneumococcal pneumonia), 482.2 (pneumonia due to Hemophilus influenzae), 482.3 (pneumonia due to Streptococcus), 482.9 (bacterial pneumonia unspecified), 483 (pneumonia due to other specified organism), 485 (bronchopneumonia, organism unspecified), 486 (pneumonia, organism unspecified). The ICD-9-CM codes were converted into the Korean Standard Classification of Diseases, Sixth Revision (KCD-6) codes, and the exclusion criteria (patients aged under 2 months diagnosed with sickle cell disease) were applied. The hospitalization rates for bacterial pneumonia were calculated per 10,000 population according to the 252 districts and were age- and sex-standardized to the Korean resident population of 2015. In addition, the hospitalization rates for three age groups were calculated: aged 0–14, 15–64, and 65 and older.

For the statistics describing geographic variation, the coefficient of variation (CV), the ratio of the 90th to the 10th percentile of the distribution of rates (P90/P10), and the systematic component of variation (SCV) were used [19–21]. The CV is the ratio of standard deviation to the mean, a normalized measure of dispersion, and the P90/P10 is a measure of dispersion in which the impact of the outliers is attenuated. As indicated by Mcpherson et al., the SCV was produced as being multiplied by 100 [19, 20].

The potential determinants of the hospitalization rates for bacterial pneumonia are as follows: First, for a socio-demographic variable 1) the deprivation index was used. It is a composite indicator of socio-economic deprivation of a region. We used the deprivation constructed on the basis of a total of 9 items: the proportion of single-person households, the proportion of female headed households, the proportion of households without car ownership, the proportion of households not living in an apartment, the proportion of households living below the minimum housing standard, the proportion of the population aged 35–64 with no high school diploma, the proportion of household heads aged 15–64 engaged in manual labor, the proportion of the population who were divorced, separated, or bereaved among those aged 15 or over, and the proportion of the population aged 65 or over [22, 23]. The score of each item was standardized by a Z-score, and its weighted values were summated as a deprivation index. The data were acquired from the Population Census 2010 of Korea.

Second, as an indicator of access to primary care, we used 2) the number of primary care physicians (per 10,000 population). We used the criteria suggested by Lee et al. which defined primary care physicians by the characteristics of patients who visited the clinics [24]. The primary care physicians were defined as physicians in the clinics where the proportion of the visits with 52 simple and minor disease groups [25] was over the average (38.3%) of total clinics [26]. To determine the influence of the number of primary care physicians as opposed to that of overall physicians, we included 3) the number of total active physicians (per 10,000 population) as an explanatory variable.

Third, regarding the density of hospital beds, we used two separate variables to differentiate the influence of hospital beds according to the size of hospitals: 4) the number of beds in hospitals with less than 300 beds for small to medium sized hospitals (per 1000 population), and 5) the number of beds in hospitals with more than 300 beds for large sized hospitals (per 1000 population). The data regarding health care resources were obtained from the Health Care Resources & Service Information Report issued by the Ministry of Health and Welfare [27].

Concerning an analysis of the factors associated with the hospitalization rates for bacterial pneumonia, we first examined the special autocorrelation in them by Moran’s *I* statistic. As substantial spatial autocorrelation was detected in the ordinary least squares (OLS) models for four outcome variables, we evaluated spatial regression models which account for spatial dependence [28].

The model took the form:

Hospitalization rate for bacterial pneumonia = β_0_ + β_1_ deprivation index score + β_2_ number of primary care physicians per 10,000 + β_3_ number of physicians per 10,000 + β_4_ number of small sized hospital beds per 1000 + β_5_ number of largel sized hospital beds per 1000 + ε with ε = λWε + ξ.

All the analyses were conducted using SAS, version 9.3 (SAS Institute, Inc., Cary, NC, USA), SPSS 23 (IBM Corporation, Armonk, NY, USA), and GeoDa, version 1. 12. 1. 161 (Spatial Analysis Lab, University of Illinois Urbana-Champaign, IL, USA).

## Results

**Fig. 1:**
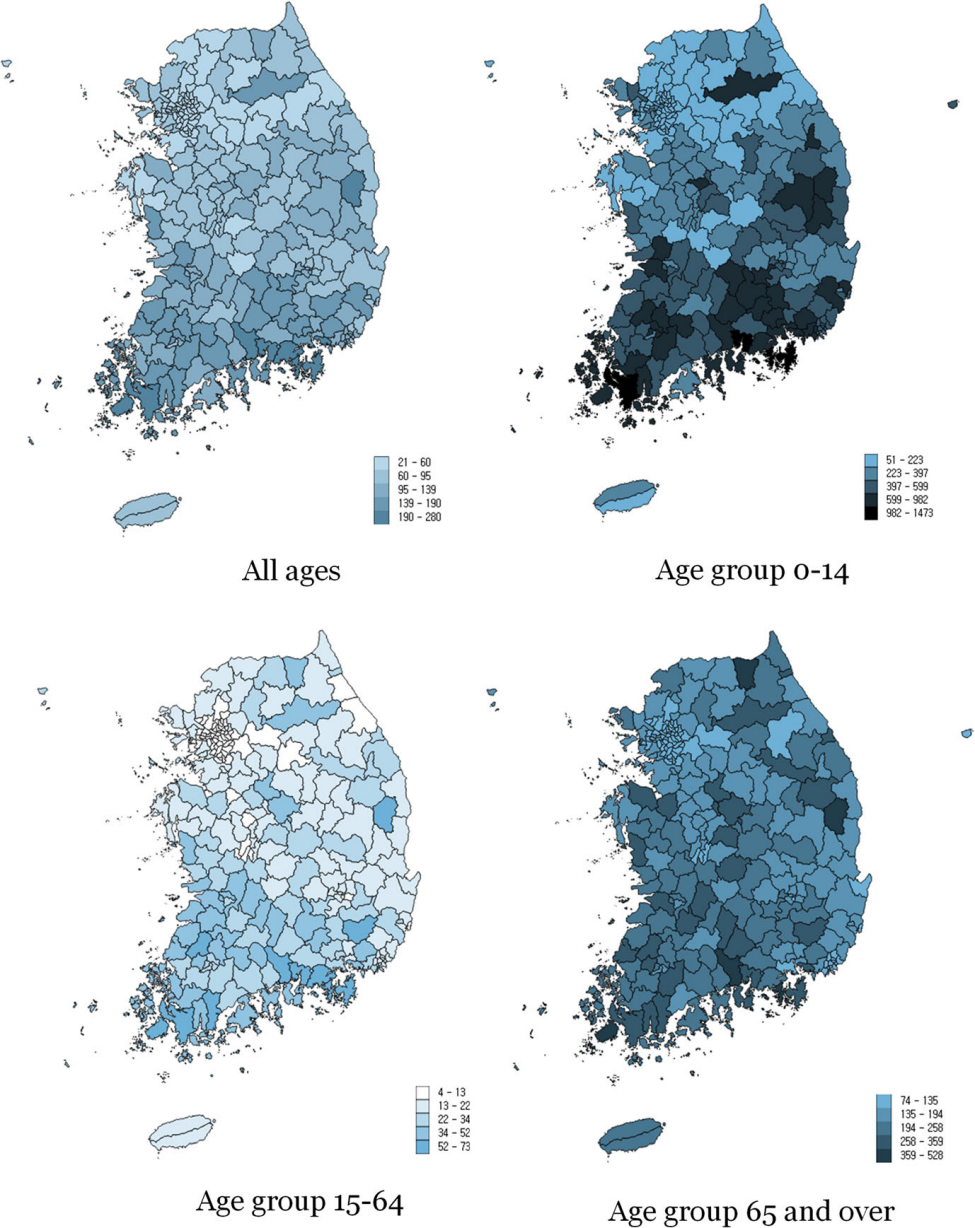
Map of preventable hospitalization rates due to bacterial pneumonia per 10,000 population in Korea in 2015. The figures were created by the author using the data provided by the National Health Insurance Service in Korea. The left upper map (for the group "All ages") was modified from the reference [30]

There were a total of 402.979 hospitalizations due to bacterial pneumonia in Korea in 2015, which was 79.1 per 10,000 population. Considering that the number of outpatient visits in 2015 amounted to 1,357,318 by the number of patients and 3,206,813 by the number of visits [29], the hospitalization for bacterial pneumonia is estimated to account for about one fifth of total incidences of bacterial pneumonia. The rate was the highest in the age group 0–14 at 325.3 per 10,000, and it was 161.5 in the age group 65 and over. The most frequent diagnosis was Pneumonia, unspecified (Table 1). The regional distribution of hospitalization rates for bacterial pneumonia is presented in Fig. 1.

**Table 1:** Absolute frequency and hospitalization rates for bacterial pneumonia per 10,000 population by age group

Diagnosis	KCD-6	Total		Aged 0 to 14		Aged 15 to 64		Aged 65 and over	
		Freq	Rate	Freq	Rate	Freq	Rate	Freq	Rate
Bacterial pneumonia (Total)		402,979	79.1	232,647	325.3	64,523	17.3	105,809	161.5
Pneumonia due to *Streptococcus pneumoniae*	J13	2790	0.5	2139	3.0	298	0.1	353	0.5
Pneumonia due to Haemophilus influenzae	J14	730	0.1	581	0.8	79	0.0	70	0.1
Pneumonia due to streptococcus, group	J15.3	60	0.0	4	0.0	31	0.0	25	0.0
Pneumonia due to Mycoplasma pneumoniae	J15.7	44,871	8.8	41,947	58.7	2199	0.6	725	1.1
Other bacterial pneumonia	J15.8	14,878	2.9	2012	2.8	4685	1.3	8181	12.5
Bacterial pneumonia, unspecified	J15.9	25,440	5.0	5204	7.3	7707	2.1	12,529	19.1
Bronchopneumonia, unspecified	J18.0	57,938	11.4	40,133	56.1	8368	2.2	9437	14.4
Other pneumonia, organism unspecified	J18.8	6650	1.3	4323	6.0	906	0.2	1421	2.2
Pneumonia, unspecified	J18.9	249,622	49.0	136,304	190.6	40,250	10.8	73,068	111.5

The CV and SCV of the hospitalization rates for bacterial pneumonia of all ages were 0.5 and 32.4 respectively (Table 2) [30]. The variation was the most marked among the people aged 0 to 14 with CV and SCV at 0.6 and 57.3, and the lowest among people aged 65 and over with CV and SCV at 0.4 and 16.7.

**Table 2:** Variation statistics of hospitalization rates for bacterial pneumonia by age group

	Mean	Max	Min	P90/P10	CV	SCV
Hospitalization rate due to bacterial pneumonia (all ages)	93.1	279.8	21.5	3.7	0.5	32.4
Hospitalization rate due to bacterial pneumonia (0–14)	386.1	1473.1	51.4	5.5	0.6	57.3
Hospitalization rate due to bacterial pneumonia (15–64)	22.9	72.5	3.5	4.5	0.6	57.8
Hospitalization rate due to bacterial pneumonia (65 and over)	182.8	527.8	74.0	2.8	0.4	16.7

We initially performed the OLS regression analyses (Additional file 1) and assessed the spatial autocorrelation in the residuals. The Moran’s *I* values were 0.369, 0.393, 0.193, and 0.215 in the OLS regression models for all ages, age group 0–14, 15–64, and 65 and over respectively. As these values suggested that there were substantial spatial autocorrelations in the residuals in all of the OLS models, we estimated spatial regression models using spatial error models (Table 3). Compared with the OLS models, the explanatory power increased in the spatial error models. The deprivation score showed positive associations, and the number of primary care physicians had a negative association with the hospitalization rates across all age groups except for the age group 0–14, where no significant association was shown. The number of beds in hospitals with less than 300 beds had a positive association with the hospitalization rates for bacterial pneumonia, and the impact was the strongest in the age group 0–14. The number of beds in hospitals with more than 300 beds was negatively associated with the hospitalization rates only in the age group 65 and over.

**Table 3:** Spatial error regression analysis of hospitalization rates for bacterial pneumonia by age group

	All ages		0–14		15–64		65 and over	
	Coefficient	SE	Coefficient	SE	Coefficient	SE	Coefficient	SE
Baseline (intercept)	111.451^***^	7.595	510.589^***^	45.852	24.396^***^	1.807	199.468^***^	10.423
Deprivation index	1.993^***^	0.744	6.268	4.113	1.514^***^	0.225	5.94^***^	1.293
Primary care physicians per 10,000 population	−4.273^**^	1.904	−7.632	10.366	−2.504^***^	0.673	−14.824^***^	3.749
Practicing physicians per 10,000 population	0.081	0.243	−0.517	1.318	0.122	0.087	0.978^**^	0.485
Hospital beds per 1000 population (< 300)	3.721^***^	0.764	11.349^**^	4.172	1.722^***^	0.26	6.911^***^	1.462
Hospital beds per 1000 population (> 300)	−1.085	1.156	−1.176	6.278	−0.766	0.417	−5.947**	2.314
Adjusted R^2^	0.674		0.643		0.575		0.525	

^**^p < 0:05 ^***^p < 0:001

## Discussion

This study investigated geographic variation in the hospitalization rates for bacterial pneumonia in Korea and its factors using the 2015 National Health Insurance Database. The overall rate for bacterial pneumonia was 79.1 per 10,000 population, and 325.3 and 161.5 for the age groups 0–14 and 65 and over respectively. The variation in the hospitalization rates for bacterial pneumonia in Korea was high with CV ranging from 0.4 to 0.6. As significant spatial autocorrelation was detected in the OLS models, we estimated spatial error models. The number of beds in hospitals with less than 300 beds had a positive association with the hospitalization rates for bacterial pneumonia across all age groups. The deprivation score showed positive associations, and the number of primary care physicians had a negative association with the hospitalization rates across all age groups except for the age group 0–14, where no significant association was shown.

The hospitalization rate for bacterial pneumonia in Korea is very high compared with other countries [8, 31–33], and the proportion of pneumonia as a cause of hospitalization in Korea is also very high compared with other countries [34–37]. This high proportion of pneumonia in hospitalization is consistent with the high proportion of pneumonia among causes of death [14]. Considering that the average health condition of the Korean people is comparatively favorable [38] and that the increase in mortality due to pneumonia is far higher than the increase in the proportion of elderly population [12, 15, 16], the high hospitalization rates for bacterial pneumonia suggest that there are factors that prompted unnecessary hospitalizations.

The high variation in the hospitalization rates for bacterial pneumonia is also suggestive of the possibility that hospitalization due to bacterial pneumonia was likely to be affected by extrinsic factors. First, the CV is at 0.6, which is markedly higher compared with that of hospitalization due to other causes [20]. This is consistent with the results in a prior study where the geographic variation in the hospitalization rates for pneumonia was higher than that for other causes [34]. The SCV also can be considered very high compared with other procedures, and this strongly indicates that the variation is due to causes which have discretionary characteristics [21]. The variation in the hospitalization rates for bacterial pneumonia was high across all age groups, but the variation in the elderly population was relatively lower than in other age groups. However, the comparatively lower variation does not necessarily mean that there is less unnecessary hospitalization, but rather it can indicate that the unnecessary utilization is universal across the regions so as to decrease the variation among the regions. The interpretation should depend on whether the hospitalization rate is reasonable or not, and our results in view of the prior studies suggest that the hospitalization rates for bacterial pneumonia are inordinately high in Korea. Therefore, the variation statistics in the hospitalization rates for bacterial pneumonia among the elderly show that the high hospitalization rate is prevalent across the regions with less variation compared with other age groups.

In the spatial error models, the deprivation index score was positively associated with the hospitalization rates due to bacterial pneumonia, which indicates the higher probability of hospitalization due to pneumonia in the more deprived regions. This is consistent with the prior studies which showed a negative association between income and the incidence of pneumonia [39–42]. Our results show that the adverse effect of socioeconomically disadvantaged conditions on pneumonia hospitalization rates also exists in the developed countries as in the case of the US [43].

The density of primary care physicians showed a negative association with the hospitalization rates for bacterial pneumonia. This result is more credible as we measured the impact of the number of primary care physicians as opposed to the total number of physicians, which showed little impact on the hospitalization rates. This is in accordance with prior studies which showed an inverse relationship between the number of primary care physicians and preventable hospitalization rates [44, 45]. And our results suggest that, in the absence of the definitive role of primary care physician supported by law or fee schedule in Korea [25], the physicians, who can be considered to perform the care close to primary care, are performing their role in preventing hospitalization due to pneumonia.

However, the deprivation index and the number of primary care physicians did not show a significant association with hospitalization rates for bacterial pneumonia in the children and adolescents (age group 0–14). The only statistically significant factor for hospitalization rate for bacterial pneumonia in age group 0–14 was the number of beds in small-sized hospitals. Although the impact of the number of beds in small-sized hospitals was shown in all age groups, it was most prominent in the children and was comparatively weak in the elderly population. This suggests that the inducement effect of the number of hospital beds on hospitalization rates is the strongest in the children. The higher influence of hospital bed density on hospitalization among the children can be more of a concern in that the hospitalization of children is not determined by their own will but by their guardians,’ and the adverse effect of unnecessary use of health care can be long-standing given their long future lifetime. Considering that the hospitalization rate due to pneumonia among children in Korea is excessively high compared with other countries [46–48], the increasing effect of the number of beds in small-sized hospitals on the hospitalization rates due to pneumonia in children suggests that it can be regarded as a main driving factor for the overuse of hospitalization. The number of beds in large-sized hospitals had a negative association with the hospitalization rates due to pneumonia among the elderly people. This result suggests a difference in the practice pattern between small- and large-sized hospitals, probably a lower tendency to induce hospitalization in large-sized hospitals. However, this might not be interpreted as the difference in the intrinsic characteristics of large- and small-sized hospitals, as there are external causes that affect their practice patterns. In Korea, large-sized hospitals are preferred by patients regardless of the severity of their illnesses [25], and the competition between small-sized hospitals gets fierce due to the sharper increase in their number compared with the large-sized hospitals. Accordingly, there can be less motivation for large-sized hospitals to induce hospitalization as there are more patients waiting to be hospitalized. Therefore, our results do not necessarily indicate that the presence of large-sized hospitals decreases the hospitalization rates for bacterial pneumonia among the elderly.

The caveat which needs to be noted in the present study is, first, regarding the definition of bacterial pneumonia. Based on the established prior literature, we included unspecified pneumonia in the category of bacterial pneumonia. This can be debatable as the unspecified ones are not necessarily caused by bacteria. However, establishing a microbial etiology is not necessarily recommended in all cases of pneumonia [49], and the result of etiologic diagnosis, if ever, is not available when the initial diagnosis is recorded. Without the results of further diagnostic tests or other definite evidence suggesting a certain type of pneumonia, unspecified pneumonia remains the major diagnosis as shown by its high proportion. The empiric treatment for most unspecified pneumonia suggests that it is considered bacterial. And, actually, viral pneumonia is rare in adults. Considering that the high proportion of unspecified pneumonia is bacterial, we decided it was more appropriate to include it, as practiced in prior studies, in bacterial pneumonia rather than to remove it. Second, this study is a cross sectional study which used a region, not an individual, as a unit of analysis. Therefore, regarding causation, our analysis needs to be reexamined in terms of temporal association and the impact of the explanatory factors on each individual hospitalization. However, our study, despite the lack of temporal association, addresses the factors which could have possibly affected the hospitalization rates for bacterial pneumonia, which are excessively high in Korea. Considering the overall socioeconomic conditions, demographic structure and health status of the Korean, this high hospitalization rate for pneumonia is hard to explain except with external factors. Our study presents a good example of how health care utilization and even prevalence of a disease can be influenced by factors which are not directly related to health.

## Conclusions

The hospitalization rates due to bacterial pneumonia in Korea are excessively high compared with other countries. This cannot simply be attributed to the high proportion of the elderly population, as the hospitalization rates were excessively high in both children and the elderly people. We found that there was a high variation in the rates of hospitalization for bacterial pneumonia in Korea. The disadvantaged socioeconomic conditions were strongly associated with increased hospitalization rates for bacterial pneumonia, and the number of primary care physicians showed an inverse association. Most of all, the number of hospital beds in small-sized hospitals had a positive association with the hospitalization rates. Our results show that pneumonia can be a major public health concern even in a developed country. And socioeconomic conditions can still be a concern in developed countries in that those countries are not totally immune from a gap in the socioeconomic conditions among the regions. Most of all, the strong impact of hospital bed supply on the hospitalization rates for pneumonia should be recognized. In particular, the fact that the number of beds in small-sized hospitals was the only factor associated with increased hospitalization rates for bacterial pneumonia among the age group 0–14 should be addressed given that patients in this age group are usually not able to exercise self-determination and that the influence of unnecessary use of health care would be long-standing. High disease burden of pneumonia in Korea, as measured by the morbidity measure and utilization, can partly be attributable to oversupply of hospital beds. In establishing policy measures for the rise in pneumonia, these factors should be taken into consideration.

## Additional file

Additional file 1: Ordinary Least Square Regression analysis of hospitalization rates for bacterial pneumonia by age group. (DOCX 13 kb)

## Abbreviations

CV: Coefficient of variation
ICD-9-CM: International Classification of Diseases, Ninth Revision, Clinical Modification
KCD-6: Korean Standard Classification of Diseases
OLS: Ordinary least squares
P90/P10: Ratio of the 90th to the 10th percentile of the distribution of rates
SCV: Systematic component of variation
