# Supplementary material for: A spatial analysis of geographic variation and factors associated with hospitalization for bacterial pneumonia in Korea
Source: BMC Pulm Med. 2019 Feb 20;19:45. doi: 10.1186/s12890-019-0798-6 (PMC6383222; doi:10.1186/s12890-019-0798-6)
Supplement: Supplementary file 1 — Ordinary Least Square Regression analysis of hospitalization rates for bacterial pneumonia by age group. (DOCX 13 kb) [file 12890_2019_798_MOESM1_ESM.docx]

Additional file 1. Ordinary Least Square Regression analysis of hospitalization rates for bacterial pneumonia by age group

|  | All ages | | 0-14 | | 15-64 | | 65 and over | |
| --- | --- | --- | --- | --- | --- | --- | --- | --- |
|  | Coefficient | SE | Coefficient | SE | Coefficient | SE | Coefficient | SE |
| Baseline (intercept) | 91.702**** | 6.256 | 358.821**** | 36.006 | 23.610**** | 1.755 | 210.936**** | 10.003 |
| Deprivation index | 3.839**** | .740 | 16.039**** | 4.261 | 1.565**** | .208 | 7.071**** | 1.184 |
| Primary care physicians per 10,000 population | -8.926** | 2.549 | -35.101** | 14.669 | -2.894**** | .715 | -19.249**** | 4.075 |
| Practicing physicians per 10,000 population | -.008 | .339 | -1.382 | 1.953 | .145 | .095 | 1.078** | .543 |
| Hospital beds per 1,000 population (<300) | 8.282**** | .944 | 40.619**** | 5.435 | 2.124**** | .265 | 7.232**** | 1.510 |
| Hospital beds per 1,000 population (>300) | -.154 | 1.617 | 8.777 | 9.306 | -.987** | .454 | -7.290** | 2.585 |
| Adjusted R^2^ | .441 |  | .322 |  | .517 |  | .435 |  |

**p<0:05 ****p<0:001
